# Anti-Inflammatory and Anti-Migratory Effects of Morin on Non-Small-Cell Lung Cancer Metastasis via Inhibition of NLRP3/MAPK Signaling Pathway

**DOI:** 10.3390/biom15010103

**Published:** 2025-01-10

**Authors:** Punnida Arjsri, Kamonwan Srisawad, Sonthaya Umsumarng, Pilaiporn Thippraphan, Songyot Anuchapreeda, Pornngarm Dejkriengkraikul

**Affiliations:** 1 Department of Biochemistry, Faculty of Medicine, Chiang Mai University, Chiang Mai 50200, Thailand; punnida.dream@gmail.com (P.A.); kamonwan.sri@cmu.ac.th (K.S.); tipprapant@gmail.com (P.T.); 2 Anticarcinogenesis and Apoptosis Research Cluster, Faculty of Medicine, Chiang Mai University, Chiang Mai 50200, Thailand; 3 Faculty of Veterinary Medicine, Chiang Mai University, Chiang Mai 50100, Thailand; sonthaya.u@cmu.ac.th; 4 Center for Research and Development of Natural Products for Health, Chiang Mai University, Chiang Mai 50200, Thailand; songyot.anuch@cmu.ac.th; 5 Department of Medical Technology, Faculty of Associated Medical Sciences, Chiang Mai University, Chiang Mai 50200, Thailand

**Keywords:** non-small-cell lung cancer, morin, inflammation, metastasis, NLRP3 inflammasome pathway

## Abstract

Non-small-cell lung cancer (NSCLC) remains the leading cause of cancer-related deaths globally, with a persistently low five-year survival rate of only 14–17%. High rates of metastasis contribute significantly to the poor prognosis of NSCLC, in which inflammation plays an important role by enhancing tumor growth, angiogenesis, and metastasis. Targeting inflammatory pathways within cancer cells may thus represent a promising strategy for inhibiting NSCLC metastasis. This study evaluated the anti-inflammatory and anti-metastatic properties of morin, a bioactive compound derived from a Thai medicinal herb, focusing on its effects on NLRP3 inflammasome-mediated pathways in an in vitro NSCLC model. The A549 and H1299 cell lines were stimulated with lipopolysaccharide (LPS) and adenosine triphosphate (ATP) to activate the NLRP3 pathway. The inhibition effects exhibited by morin in reducing pro-inflammatory secretion in LPS- and ATP-stimulated NSCLC cells were assessed by ELISA, while wound healing and trans-well invasion assays evaluated its impact on cell migration and invasion. RT-qPCR measurement quantified the expression of inflammatory genes, and zymography and Western blotting were used to examine changes in invasive protein levels, epithelial-to-mesenchymal transition (EMT) markers, and underlying molecular mechanisms. Our findings demonstrated the significant ability of morin to decrease the production of IL-1β, IL-18, and IL-6 in a dose-dependent manner (*p* < 0.05), as well as suppress NSCLC cell migration and invasion. Morin downregulated invasive proteins (MMP-2, MMP-9, u-PAR, u-PA, MT1-MMP) and EMT markers (fibronectin, N-cadherin, vimentin) (*p* < 0.01) while also reducing the mRNA levels of NLRP3, IL-1β, IL-18, and IL-6. Mechanistic investigations revealed that morin suppressed NLRP3 inflammasome activity and inactivated MAPK pathways. Specifically, it decreased the expression of NLRP3 and ASC proteins and reduced caspase-1 activity, while reducing the phosphorylation of ERK, JNK, and p38 proteins. Collectively, these findings suggest that morin’s inactivation of the NLRP3 inflammasome pathway could offer a novel therapeutic strategy for counteracting pro-tumorigenic inflammation and metastatic progression in NSCLC.

## 1. Introduction

Non-small-cell lung cancer (NSCLC) remains a significant public health challenge due to its high incidence, high metastasis rate, treatment resistance, and elevated mortality. With only 15.9% of patients surviving 5 years or longer post-diagnosis [1], NSCLC frequently metastasizes to critical organs, with a high likelihood of spreading to the brain (47%), bones (36%), and liver (22%). Additionally, reports indicate that NSCLC can metastasize to the thoracic cavity (11%), adrenal glands (15%), and distant lymph nodes (10%) [2]. Metastasis remains a primary driver of poor outcomes and mortality in NSCLC [3,4], making it essential to unravel the cellular mechanisms that underlie NSCLC progression.

Inflammation is a key factor in cancer development, not only initiating tumorigenesis but also fostering progression. It enables the acquisition of the six hallmarks of cancer, thus facilitating tumor growth, survival, and metastasis spread [5,6,7]. Recently, inflammasome-mediated inflammation has gained significant attention in cancer research. Inflammasomes are intracellular protein complexes that initiate inflammatory responses upon exposure to numerous stimuli [8]. Among the over 20 identified inflammasomes, the NLRP3 inflammasome has been extensively reported on in previous studies and is recognized for its important role in inflammation. The NLRP3 inflammasome, composed of the NOD-like receptor NLRP3, the adaptor protein ASC (apoptosis-associated speck-like protein containing a caspase recruitment domain), and pro-caspase-1, is activated through a two-step process. The first step involves stimulating the expression of the NLRP3 molecule along with the pro-forms of inflammatory cytokines (IL-1β and IL-18) in response to signals from danger-associated molecular patterns (DAMPs) or pathogen-associated molecular patterns (PAMPs), such as lipopolysaccharide (LPS). The second step for activating NLRP3 requires a trigger, such as adenosine 5′-triphosphate (ATP), silica, or monosodium urate (MSU), which promotes the assembly of NLRP3, ASC, and pro-caspase-1. This assembly cleaves pro-IL-1β and pro-IL-18, releasing these active inflammatory cytokines [9,10].

Multiple earlier studies have reported that the activation of the NLRP3 inflammasome can promote cancer migration and metastasis across various types. For instance, in melanoma, active NLRP3 inflammasomes drive autoinflammation via caspase-1 and the release of IL-1β and IL-18 cytokines [11]. Elevated NLRP3 expression in oral squamous cell carcinoma is associated with tumor growth, epithelial-to-mesenchymal transition (EMT), and metastasis [12,13]. In breast cancer, the activation of NLRP3 stimulates the secretion of IL-1β, which promotes EMT and facilitates metastasis [14]. The suppression of NLRP3 inflammasome activation has demonstrated potential in reducing NSCLC cell proliferation and metastasis [15,16], positioning it as a promising therapeutic target in NSCLC treatment.

Morin (2′,3,4′,5,7-pentahydroxyflavonone) is a yellow flavonoid from *Maclura cochinchinensis* (Kae-Lae), a medicinal herb traditionally used in Thai medicine [17]. Morin is known for its wide range of biological activities, including antioxidant, anti-inflammatory, anti-mutagenic, and anti-carcinogenic effects, particularly in liver cancer models [18,19]. Its favorable safety profile makes it an attractive therapeutic candidate [20]. Morin has been utilized in Traditional Chinese Medicine (TCM) since medieval times, with formulations containing this compound documented in historical practices. A search of the TCM-ID database using the term ’MORIN’ identified 21 entries listing traditional Chinese formulations that include morin as a component [18]. Numerous research studies and clinical investigations have highlighted the pharmacological potential of morin, demonstrating its biological activities, including antidiabetic, anti-arthritic, cardioprotective, neuroprotective, nephroprotective, and hepatoprotective effects [20]. Morin has demonstrated anti-inflammatory efficacy, notably in the chronic phase of trinitrobenzenesulfonic-acid-induced colitis in rats [21], and has been demonstrated to suppress airway inflammation in ovalbumin-induced asthma by modulating MAPK signaling [22]. Furthermore, morin inhibits growth and invasion in the MDA-MB-231 cell line, a metastatic breast cancer model [23]. However, its effects on inflammation-mediated cancer metastasis are not yet fully understood, underscoring the need to explore morin’s potential in preventing inflammation-driven cancer spread.

In our study, we demonstrated the in vitro effects of morin on inflammation-related cancer metastasis in NSCLC using the A549 and H1299 cell lines primed with LPS and ATP to activate the NLRP3 inflammasome. Our study aimed to assess the anti-inflammation, anti-migration, and anti-invasion properties of morin. Our results demonstrated that morin effectively suppressed NSCLC metastasis by inhibiting NLRP3 inflammasome activation and the MAPK signaling pathway. These findings provide scientific evidence supporting morin’s potential as a therapeutic agent targeting the NLRP3 inflammasome in NSCLC.

## 2. Materials and Methods

### 2.1. Chemical and Reagents

The morin compound was acquired from ^©^Tokyo Chemical Industry Co., Ltd. (Chuo-ku, Tokyo, Japan). The protease inhibitor cocktail, RIPA lysis buffer (modified radioimmunoprecipitation assay), enhanced chemiluminescence (ECL) reagent, and Commasie Plus™ protein assay reagent were obtained from Thermo Fisher Scientific (Rockford, IL, USA). The sulforhodamine B (SRB) reagent, lipopolysaccharide (LPS), adenosine triphosphate (ATP), and anti-β-actin primary antibody were purchased from Sigma-Aldrich (St. Louis, MO, USA). The media (Dulbecco’s Modified Eagle Medium; DMEM), penicillin–streptomycin, and Fetal bovine serum (FBS) were bought from Gibco BRL Company (Grand Island, NY, USA). Primary antibodies for Western blot analysis to detect NLRP3, ASC, caspase-1, invasive proteins (u-PA, u-PAR, and MT1-MMP), EMT markers (fibronectin, N-cadherin, and vimentin), and MAPK signaling pathway (phospho-ERK, ERK, phospho-JNK, JNK, phospho-p38, and p38) protein expression, and the secondary antibodies, namely, anti-rabbit or anti-mouse IgG conjugated with horseradish peroxidase, were obtained from Cell Signaling Technology (Beverly, MA, USA). The reagent QIAzol lysis was acquired from Qiagen (Valencia, CA, USA). The SensiFAST SYBR Lo-ROX Kit was bought from Meridian Bioscience^®^ (Cincinnati, OH, USA). The qPCR Master Mix (ReverTra Ace^®^) was obtained from Toyobo Co., Ltd. (Osaka, Japan).

### 2.2. Cell Cultures

In our study, we used the A549 (CCL-185™) and H1299 (CRL-5803™) cell lines, which are NSCLC cell line models. These cell lines were acquired from American Type Culture Collection (ATCC) (Manassas, VA, USA). NSCLC cell lines remained in the culture medium (DMEM). The cells were maintained with 50 IU/mL of penicillin, 50 μg/mL of streptomycin, and 10% FBS. The cells were cultured in a 5% CO_2_ humidified incubator at 37 °C. The cells were harvested at 70–80% confluency and seeded for each experiment.

### 2.3. The Sulforhodamine B (SRB) Assay

The cell viability assay was used to investigate the effects of the morin compound on A549 and H1299 cell viability through SRB dye staining [24]. A549 and H1299 cells (3 × 10^3^ cells/well) were plated into 96-well plates and maintained overnight at 37 °C in 5% CO_2_. Varying concentrations of the morin compound (0–165 μM) were then added, and cells were cultured for 24 and 48 h. To each well, 10% (*w*/*v*) trichloroacetic acid (100 μL) was added, and the cells were fixed in a refrigerator (4 °C) for 1 h. After incubation, the solution was discarded, and the wells were washed gently with tap water. Subsequently, 0.054% (*w*/*v*) sulforhodamine B dye (100 μL) was added to stain proteins for 30 min at room temp. After staining, the SRB dye was cleansed, and the wells were rinsed with 1% (*v*/*v*) acetic acid and allowed to air-dry at room temp. The stained dye was solubilized in 10 mM Tris-base (pH 10.5) (100 μL). After that, absorbance was detected by a microplate reader at 510 nm. Cell viability was calculated as a percentage of the control.

### 2.4. Measurement of Pro-Inflammatory Cytokines Released by Enzyme-Linked Immunosorbent Assay (ELISA)

Using an ELISA kit, pro-inflammatory cytokine levels, including IL-6, IL-1β, and IL-18, were measured in the cell culture medium according to the manufacturer’s protocol (Biolegend, San Diego, CA, USA), as previously outlined [25]. A549 and H1299 cells were plated in a six-well plate at a density of 2 × 10^5^ cells/well. After incubation overnight, the cells were treated with varying doses of the morin compound (0–132 μM) for 24 h and then were induced by LPS—lipopolysaccharide—at 1 mg/mL for 6 h, followed by 5 nM of ATP—adenosine triphosphate—for an additional 30 min [26,27]. Then, the supernatant medium of each condition was collected, and following the protocol, absorbance was detected by a microplate reader at 450 and 570 nm. The release levels of pro-inflammatory cytokines were quantified by comparing the results to a standard curve.

### 2.5. Wound–Scratch Assay

To evaluate the migratory ability of NSCLC cells, a wound–scratch assay was performed following the established protocol [28]. Briefly, A549 and H1299 cells were plated at 2 × 10^5^ cells/well (6-well plate). To eliminate the influence of cell proliferation factors, the A549 or H1299 cells were cultured overnight in DMEM with 0.5% FBS. The A549 and H1299 cells were cultured to 90–100% confluence, and a wound was generated using a 200 μL pipette tip. Subsequently, the cells were treated with varying doses of the morin compound (0–132 μM) for 24 h. Then, the cells were induced by LPS at 1 mg/mL for 6 h, followed by 5 nM of ATP for an additional 30 min. At 0 and 24 h, images were captured in the same fields using 100× magnification on a phase-contrast microscope (Nikon Eclipse TS100, Nikon Instruments Inc., Tokyo, Japan). ImageJ 1.410 software (“Wound Healing Size” plugin) was used to analyze the wound area [29].

### 2.6. Cell Invasion Assay

To assess the effect of the morin compound on the invasive abilities of A549 and H1299 cells, trans-well invasion assays were conducted following a previously established protocol [30]. Polyvinylpyrrolidone-free polycarbonate filters with an 8 μm pore size, purchased from BD Biosciences (Franklin Lakes, NJ, USA), were used in this study. The filters were coated with Matrigel matrix at 15 μg per filter. To eliminate the effect of cell proliferation on data interpretation, 5 × 10^4^ A549 and H1299 cells were seeded into each upper chamber in DMEM containing 0.1% FBS, along with varying concentrations of the morin compound (0–132 μM). The lower chamber was filled with DMEM containing 10% FBS, which acts as a chemoattractant. The cells were induced with LPS at 1 mg/mL for 24 h of incubation. After 6 h of LPS induction, 5 nM of ATP was added to the upper chamber for an additional 30 min. The cells that invaded through the membrane to the lower surface of the chamber were fixed with 95% ethanol for 5 min. The membranes were stained with crystal violet (0.5%) for 30 min. The stained cells were captured using a phase-contrast microscope (Nikon Eclipse TS100, Nikon Instrument Inc.) The areas occupied by cells were measured using ImageJ software (version 1.410) to quantify invasion activity.

### 2.7. Gelatin Zymography Assay

To assess the effects of the morin compound on MMP-2 and MMP-9 secretion by the A549 and H1299 cells, a gelatin zymography assay was performed. Firstly, the A549 and H1299 cells were plated at 2 × 10^5^ cells/well and cultured overnight. Next, the cells were treated with varying doses of the morin compound (0–132 μM) and incubated for 24 h. They were then induced with LPS at 1 mg/mL for 6 h, followed by 5 nM of ATP for an additional 30 min. Following incubation, the supernatant medium was collected for analysis. Proteins were separated by SDS-PAGE under non-reducing conditions using a 10% polyacrylamide gel containing 0.1 mg/mL gelatin. Following electrophoresis, the gel was washed with 2.5% Triton X-100 and incubated for 1 h. The gelatinase buffer (10 mM CaCl₂, 50 mM Tris-HCl, and 200 mM NaCl, pH 7.4) was used to activate MMP enzyme activity at 37 °C for 24 h. Next, the gel was stained with 0.1% (*w*/*v*) Coomassie Brilliant Blue dye and then destained with a solution containing methanol, acetic acid, and water in a 50:10:40 ratio. Gelatinolytic activity was quantified by analyzing band intensity with ImageJ software version 1.410.

### 2.8. Measurement of Pro-Inflammatory Cytokines and NLRP3 mRNA Levels by Reverse Transcription–Quantitative Real-Time Polymerase Chain Reaction (RT-qPCR) Analysis

To measure the expressions of pro-inflammatory cytokine genes (*NLRP3*, *IL-1β*, *IL-18*, and *IL-6*), NSCLC cell lines were treated with the morin compound (0–132 μM) for 24 h. They were then induced with LPS at 1 mg/mL for 6 h, and then 5 nM of ATP was added for an additional 30 min. Using the QIAzol^®^ lysis reagent, total mRNA was extracted. A NanoDrop™ 2000/2000c Spectrophotometer (Thermo Fisher Scientific, Waltham, MA, USA) was used for determining the concentration and purity of total RNA. Reverse transcription was performed using a Mastercycler^®^ Nexus Gradient machine (Eppendorf, GA, Germany) to synthesize cDNA. Quantitative real-time PCR was conducted on an ABI 7500 Fast and 7500 Real-Time PCR machine (Thermo Fisher Scientific, Waltham, MA, USA). The expression of NLRP3 and cytokine genes was analyzed by QuantStudio 6 Flex real-time PCR system software v1.0 (Applied Biosystems, Waltham, MA, USA) and the 2^−ΔΔCT^ method. Moreover, GAPDH was used to normalize the control samples before calculating the results.

The sequences of primers used in our study are listed below: NLRP3 forward: 5′-AAC ATG CCC AAG GAG GAA GA-3′, reverse: 5′-GGC TGT TCA CCA ATC CAT GA-3′; IL-1β forward: 5′-TGC TCA AGT GTC TGA AGC AG-3′, reverse: 5′-TGG TGG TCG GAG ATT CGT AG-3′; IL-18 forward: 5′-TCG GGA AGA GGA AAG GAA CC-3′, reverse: 5′-TTC TAC TGG TTC AGC AGC CA-3′; IL-6 forward: 5′-CAA TCT GGA TTC AAT GAG GAG AC-3′, reverse: 5′-TTT TTC TGC AGG AAC TGG ATC AG-3′; and GAPDH forward: 5′-TCA ACA GCG ACA CCC AC-3′, reverse: 5′-GGG TCT CTC TCT TCC TCT TGT G-3′ (Humanizing Genomics Macrogen, Geumcheon-gu, Seoul, Republic of Korea) [31,32,33,34].

### 2.9. Western Blot Analysis

To examine the inhibitory mechanism of the morin compound on inflammasome machinery proteins in LPS+ATP-stimulated inflammation, NSCLC cells were pre-treated with the morin compound (0–132 μM) for 24 h. They were then induced with LPS at 1 mg/mL for 6 h, followed by 5 nM of ATP for an additional 30 min. The protein concentrations of the samples were measured using the Bradford assay after they were collected and lysed using the RIPA buffer. The whole-cell lysates were separated on 8–12% SDS-PAGE gel. After separation, the sample proteins were transferred onto nitrocellulose membranes. After this, 3% of bovine serum albumin (BSA) was used for blocking for 1 h and then washed with 0.5% TBS-Tween before being incubated with the primary antibody overnight at 4 °C. Following this, membranes were washed five times with 0.5% TBS-Tween and incubated with the secondary antibody at room temperature for 2 h. The membranes were washed with 0.5% TBS-Tween (four times), and the proteins were observed by a chemiluminescent detection system and visualized on an iBright™ CL-1500 imaging system (Thermo Fisher Scientific, Waltham, MA, USA). Anti-β-actin antibody was used to confirm protein loading consistency in each lane. Band intensities were quantified using ImageJ software version 1.410.

### 2.10. Statistical Analysis

All experiments were performed in triplicate independent trials to ensure reproducibility. Statistical analyses were performed using GraphPad Prism software, version 8.0., based on data from three independent experiments. Data analysis, including results from the cell viability assay, ELISA, RT-qPCR, and Western blot assay, was carried out using the independent *t*-test and one-way ANOVA with Dunnett’s test. The results are presented as the mean ± standard deviation from three independent experiments. Statistical significance was established at * *p* < 0.05, ** *p* < 0.01, and *** *p* < 0.001.

## 3. Results

### 3.1. Effects of Morin on NSCLC Cell Viability

**Figure 1:**
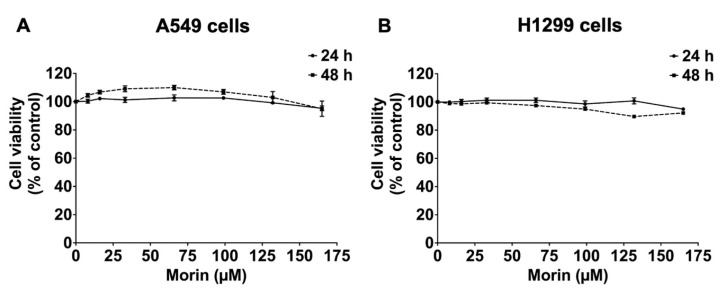
Effects of morin on the viability of NSCLC cells—A549 (**A**) and H1299 (**B**) cells—as determined by the SRB assay. NSCLC cells were treated with varying doses of morin (0–165 μM) for 24 and 48 h. These results are reported as the mean ± standard deviation.

Before the anti-inflammatory effects of morin were evaluated, its effects on the viability of NSCLC cell lines (A549 and H1299 cells) were assessed. Cell viability after treatment with varying doses of morin (0–165 μM, for 24 and 48 h) was demonstrated by the SRB assay. Figure 1A,B illustrate that morin shows no significant effects on cell viability in either A549 and H1299 cells within this dose range and duration. Based on these findings, morin concentrations ranging from 0 to 132 were selected for subsequent experiments. These results suggest that morin is not harmful to NSCLC cells, including both A549 and H1299 cells, under the conditions tested, allowing for further investigation of its biological effects without inducing cytotoxicity.

### 3.2. Effect of Morin on Anti-Inflammatory Properties in LPS+ATP-Stimulated Cytokine Secretion of NSCLC Cells

**Figure 2:**
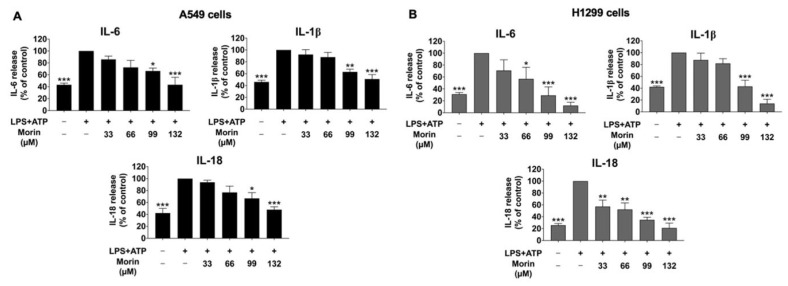
The suppressive effects of morin treatment on pro-inflammatory cytokine secretion in LPS+ATP-stimulated NSCLC cells: A549 (**A**) and H1299 (**B**) cells. NSCLC cells were pre-treated with varying doses of morin (0–132 μM) for 24 h, followed by induction with LPS (1 mg/mL) for 6 h and subsequent stimulation with ATP (5 nM) for an additional 30 min. The cytokines in the supernatant medium (IL-6, IL-1β, and IL-18) were measured using ELISA. These data are presented as percentages relative to the LPS+ATP-stimulated group, which is defined as 100%. These results are reported as the mean ± standard deviation, with * *p* < 0.05, ** *p* < 0.01, and *** *p* < 0.001, compared to the control group (LPS+ATP-stimulated group).

To investigate the suppressive effects of morin on LPS+ATP-stimulated pro-inflammatory cytokine secretion in NSCLC cell lines, ELISA was performed. The levels of pro-inflammatory cytokines, including IL-6, IL-1β, and IL-18, released into the culture medium of NSCLC cells were detected. The results demonstrated that the stimulation of NSCLC cells with LPS and ATP significantly enhanced the secretion of IL-6, IL-1β, and IL-18 compared to in the non-stimulated control group (*p* < 0.001) in both A549 and H1299 cells, as illustrated in Figure 2. However, morin treatment led to a significant and dose-dependent reduction in the release levels of these cytokines in NSCLC cells compared to in the LPS+ATP-stimulated control group (*p* < 0.001) (Figure 2A,B). These findings suggest that morin exhibits potent anti-inflammatory properties by decreasing the secretion of pro-inflammatory cytokines, including IL-6, IL-1β, and IL-18, in the culture supernatant of LPS+ATP-stimulated NSCLC cells.

### 3.3. Effects of Morin on Anti-Migration Properties in LPS+ATP-Stimulated NSCLC Cells

**Figure 3:**
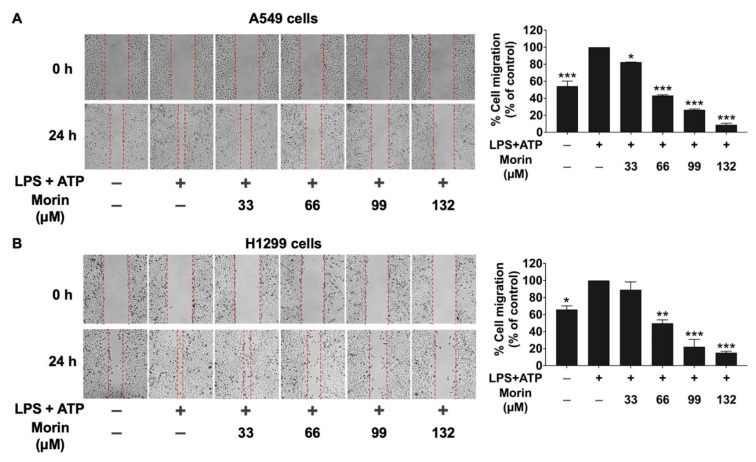
The anti-migration effect of morin on LPS+ATP-stimulated NSCLC cells: A549 (**A**) and H1299 (**B**) cells. The anti-migration properties of morin (0–132 μM) were evaluated using a scratch assay. NSCLC cells were primed with LPS (1 mg/mL) for 6 h, followed by stimulation with ATP (5 nM) for an additional 30 min. The images were captured after the initial scratch at 0 and 24 h. Migrated cells were visualized using phase-contrast microscopy and quantified with ImageJ software. These results are reported as the mean ± standard deviation, with * *p* < 0.05, ** *p* < 0.01, and *** *p* < 0.001, compared to the control group (LPS+ATP-stimulated group).

The inflammatory response of lung cancer cells via the NLRP3 inflammasome pathway is known to enhance cancer cell migration and invasion. To investigate the effects of morin on lung cancer cell migration inhibition, wound healing assays were performed on LPS+ATP-stimulated NSCLC cells. The results showed that LPS+ATP-stimulated NSCLC cells exhibited significantly increased migration compared to non-stimulated controls (Figure 3). However, morin treatment led to a significant and dose-dependent decrease in cell migration in both LPS+ATP-stimulated NSCLC cell lines (Figure 3A,B), while cells treated with morin alone showed no inhibitory effect on cell migration (Supplementary Materials Figure S1).

### 3.4. Effects of Morin on Anti-Invasion Properties in LPS+ATP-Stimulated NSCLC Cells

**Figure 4:**
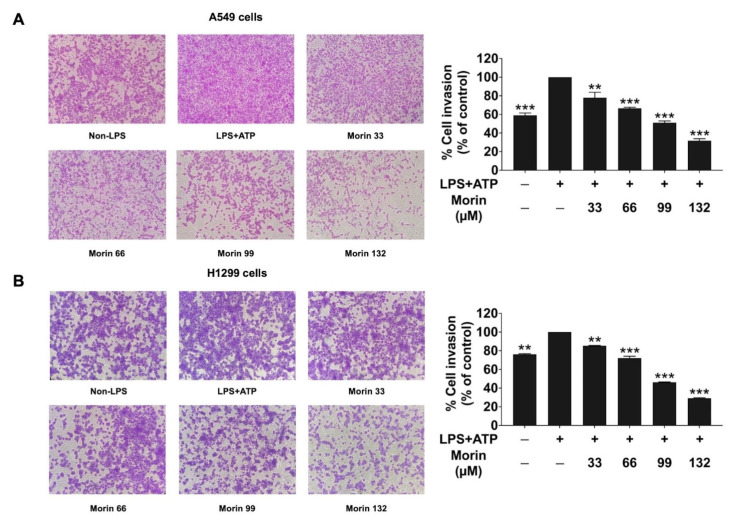
Effects of morin on the invasion of LPS+ATP-stimulated NSCLC cells: A549 (**A**) and H1299 (**B**) cells. A trans-well invasion assay was conducted to assess the anti-invasive activity of morin (0–132 μM) against LPS+ATP-stimulated NSCLC cells. NSCLC cells were primed with LPS (1 mg/mL) for 6 h, followed by stimulation with ATP (5 nM) for an additional 30 min. Invading cells were visualized at 0 and 24 h using phase-contrast microscopy and quantified with ImageJ software. These results are reported as the mean ± standard deviation, with ** *p* < 0.01 and *** *p* < 0.001, compared to the control group (LPS+ATP-stimulated group). Representative images were visualized through ×100 magnitude, captured with a Nikon Eclipse TS100 digital camera (Nikon, Tokyo, Japan).

The impact of morin on the invasion of LPS+ATP-stimulated NSCLC cells was assessed using a trans-well invasion assay. The findings revealed that stimulation with LPS+ATP significantly increased the invasion of NSCLC cells (A549 and H1299 cells) compared to non-stimulated controls (Figure 4). However, morin treatment led to a significant and dose-dependent inhibition of the invasion of NSCLC cells, as evidenced by the trans-well assay results. These findings suggest that LPS+ATP stimulated the inflammatory response through the NLRP3 inflammasome pathway, enhancing the invasive capability of NSCLC. Moreover, the data suggest that morin exhibits strong anti-metastasis properties in LPS+ATP-stimulated NSCLC cells by effectively inhibiting both cell migration and invasion.

### 3.5. Effects of Morin on the Expression of Invasive Proteins and Mesenchymal Markers in LPS+ATP-Stimulated NSCLC Cells

**Figure 5:**
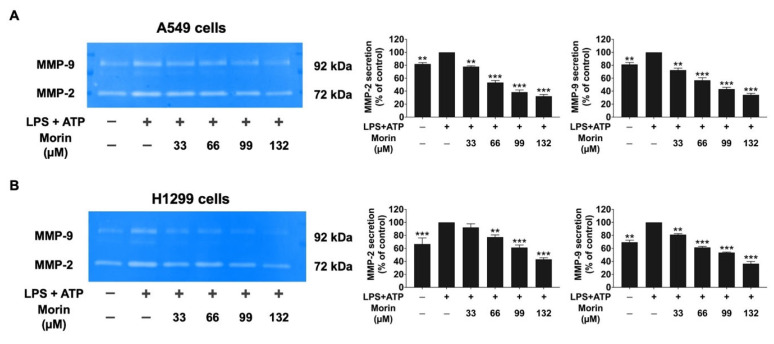
The inhibitory effects of morin on the secretion of MMP-2 and MMP-9 in LPS+ATP-stimulated NSCLC cells: A549 (**A**) and H1299 (**B**) cells. NSCLC cells were treated with various doses of morin (0–132 μM) for 24 h. NSCLC cells were primed with LPS (1 mg/mL) for 6 h, followed by stimulation with ATP (5 nM) for an additional 30 min. The supernatants from the culture were collected, and MMP-2 and MMP-9 secretion levels were assessed using a gelatin zymography assay. These results are reported as the mean ± standard deviation, with ** *p* < 0.01 and *** *p* < 0.001, compared to the control group (LPS+ATP-stimulated group).

**Figure 6:**
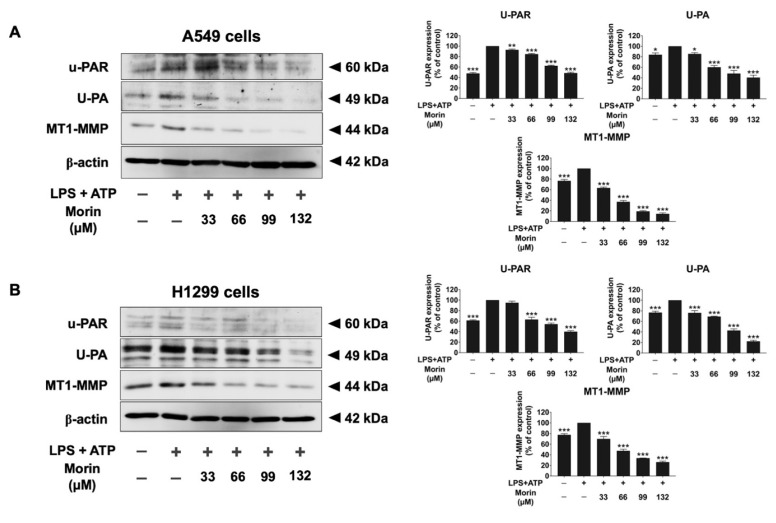
The suppressive effects of morin on invasive protein levels in LPS+ATP-stimulated NSCLC cells: A549 (**A**) and H1299 (**B**) cells. NSCLC cells were treated with morin at concentrations ranging from 0 to 132 μM for 24 h. NSCLC cells were primed with LPS (1 mg/mL) for 6 h, followed by stimulation with ATP (5 nM) for an additional 30 min. Western blotting and band density measurements were used to analyze the effects of morin on the expression of invasive proteins in NSCLC cells. These data are presented as percentages relative to the LPS+ATP-stimulated group, which is defined as 100%. These results are reported as the mean ± standard deviation, with * *p* < 0.05, ** *p* < 0.01, and *** *p* < 0.001, compared to the control group (LPS+ATP-stimulated group). Original Western blot images can be found in Figures S2 and S3.

**Figure 7:**
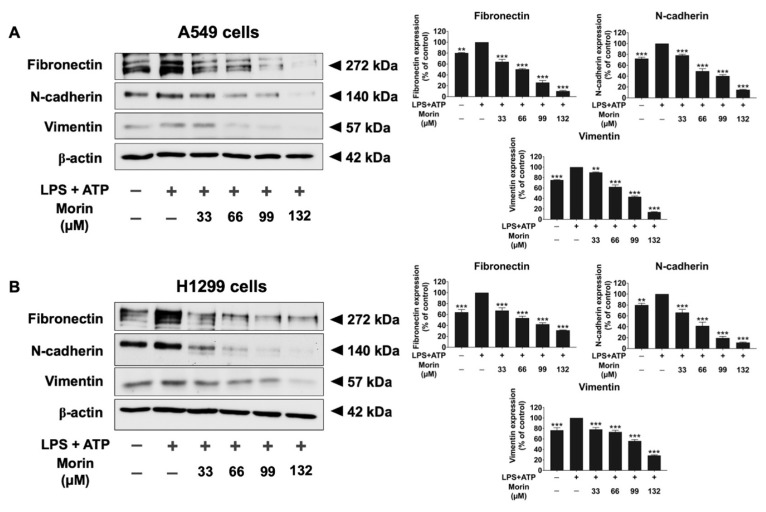
The suppressive effects of morin on mesenchymal markers in LPS+ATP-stimulated NSCLC cells: A549 (**A**) and H1299 (**B**) cells. NSCLC cells were treated with morin at doses ranging from 0 to 132 μM for 24 h. NSCLC cells were primed with LPS (1 mg/mL) for 6 h, followed by stimulation with ATP (5 nM) for an additional 30 min. Western blotting and band density measurements were used to investigate the inhibitory effects of morin on the expression of mesenchymal markers in NSCLC. These data are presented as percentages relative to the LPS+ATP-stimulated group, which is defined as 100%. These results are reported as the mean ± standard deviation, with ** *p* < 0.01, and *** *p* < 0.001, compared to the control group (LPS+ATP-stimulated group). Original Western blot images can be found in Figures S4 and S5.

To investigate the mechanism by which morin inhibits NSCLC migration and invasion, we examined the expression of invasive proteins (MMP-2, MMP-9, u-PA, u-PAR, and MT1-MMP) and EMT markers (fibronectin, N-cadherin, and vimentin) in LPS+ATP-stimulated NSCLC cells. A gelatin zymography assay was used to measure the secretion levels of MMP-2 and MMP-9, as shown in Figure 5. LPS+ATP stimulation significantly increased the release of MMP-2 and MMP-9 in NSCLC cells compared to non-stimulated controls. However, morin demonstrated significant, dose-dependent inhibition of MMP-2 and MMP-9 secretion in both cell lines (*p* < 0.001), as illustrated in Figure 5A,B. Western blot analysis was used to assess the inhibitory effects of morin on invasive protein expression levels (u-PA, u-PAR, and MT1-MMP). The results revealed that LPS+ATP-stimulated NSCLC cells showed significantly higher expression levels of u-PA, u-PAR, and MT1-MMP compared to non-stimulated controls. Treatment with morin significantly decreased the expression of these invasive proteins in a dose-dependent manner in NSCLC cells (*p* < 0.001), as illustrated in Figure 6A,B (Supplementary Materials Figures S2 and S3). Furthermore, the inhibitory effects of morin on mesenchymal markers in LPS+ATP-stimulated NSCLC cells were determined by Western blot analysis. Our findings reported that LPS+ATP stimulation led to increased expression of fibronectin, N-cadherin, and vimentin in both A549 and H1299 cells compared to non-stimulated controls. Treatment with morin significantly reduced the expression of these mesenchymal markers in a dose-dependent manner (*p* < 0.001), as illustrated in Figure 7A,B (Supplementary Materials Figures S4 and S5). Our investigation suggests that morin effectively inhibits the EMT process in NSCLC cells by decreasing the expression of mesenchymal markers. Overall, morin suppresses NSCLC metastasis by inhibiting ECM degradation via decreased MMP-2 and MMP-9 secretion, the downregulation of u-PA, u-PAR, and MT1-MMP expression, and the inhibition of mesenchymal marker expression.

### 3.6. Inhibitory Effects of Morin on the Expression of Pro-Inflammatory Cytokines and NLRP3 Genes in LPS+ATP-Stimulated NSCLC Cells

**Figure 8:**
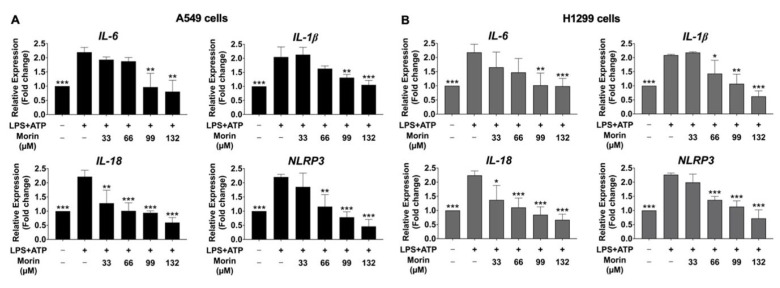
The suppressive effects of morin on pro-inflammatory cytokine (*IL-6*, *IL-1β*, *IL-18*, and *NLRP3*) gene expression in LPS+ATP-stimulated NSCLC cells: A549 (**A**) and H1299 (**B**) cells. NSCLC cells were treated with morin (0–132 μM) for 24 h. Following treatment, the cells were primed with LPS (1 mg/mL) for 6 h, followed by stimulation with ATP (5 nM) for an additional 30 min. RT-qPCR was used to analyze the mRNA expression levels of IL-6, IL-1β, IL-18, and NLRP3. These results are reported as the mean ± standard deviation, with * *p* < 0.05, ** *p* < 0.01, and *** *p* < 0.001, compared to the control group (LPS+ATP-stimulated group).

Our study aimed to determine the inhibitory effects of morin on the gene expression of NLRP3, as well as pro-inflammatory cytokines (IL-6, IL-1β, and IL-18), in LPS+ATP-stimulated NSCLC cell lines, specifically A549 and H1299 cells, using RT-qPCR analysis to measure mRNA expression. The results exhibited that LPS+ATP stimulation significantly enhanced the mRNA levels of the *NLRP3* gene, as well as the cytokine genes *IL-6*, *IL-1β*, and *IL-18*, compared to the non-stimulated group (*p* < 0.001) in NSCLC cells, as illustrated in Figure 8. Conversely, morin treatment led to a significant and dose-dependent suppression of the mRNA expression of IL-6, IL-1β, IL-18, and NLRP3 in LPS+ATP-induced NSCLC cells compared to in the LPS+ATP-stimulated group (*p* < 0.001) (Figure 8A,B) of NSCLC cells. These findings suggest that morin has potential as an inhibitor of the NLRP3 inflammasome cascade in LPS+ATP-stimulated inflammation in NCSLC cell lines.

### 3.7. Effects of Morin on Expression of Protein-Related NLRP3 Inflammasome Pathway in LPS+ATP-Stimulated NSCLC Cells

**Figure 9:**
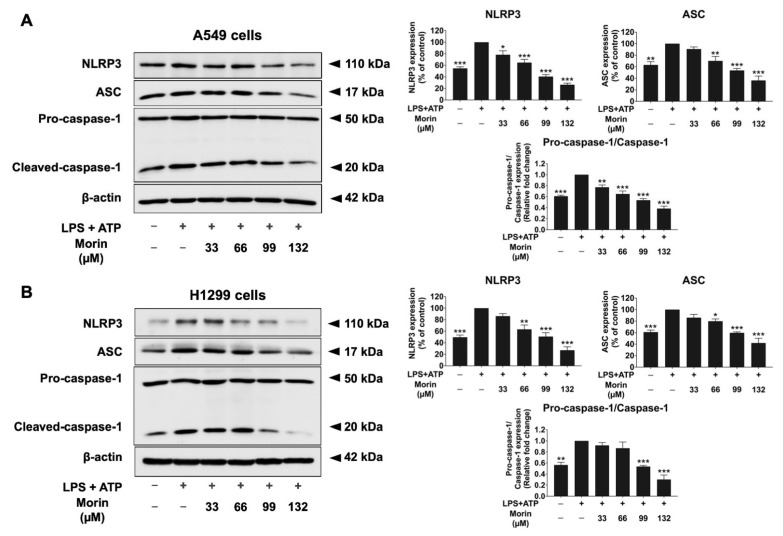
The suppressive effects of morin on the NLRP3 inflammasome pathway in LPS+ATP-stimulated NSCLC cells: A549 (**A**) and H1299 (**B**) cells. NSCLC cells were treated with morin (0–132 μM) for 24 h. Following treatment, the cells were stimulated with LPS (1 mg/mL) for 6 h, followed by stimulation with ATP (5 nM) for an additional 30 min. Western blotting and band density measurements were used to analyze the effects of morin on the expression of the NLRP3 inflammasome pathway (NLRP3, ASC, and pro-caspase-1 and cleaved-caspase-1 proteins) in NSCLC cells. These data are presented as percentages relative to the LPS+ATP-stimulated group, which is defined as 100%. These results are reported as the mean ± standard deviation, with * *p* < 0.05, ** *p* < 0.01, and *** *p* < 0.001, compared to the control group (LPS+ATP-stimulated group). Original Western blot images can be found in Figures S6 and S7.

NLRP3, ASC, pro-caspase-1, and cleaved-caspase-1 are components of the NLRP3 inflammasome pathway. When the NLRP3 inflammasome pathway is activated, NLRP3 interacts with ASC and recruits pro-caspase-1, leading to its activation into cleaved-caspase-1. This activation subsequently triggers the release of the IL-1β and IL-18 pro-inflammatory cytokines. To evaluate the effects of morin on the NLRP3 inflammasome pathway in LPS+ATP-stimulated NSCLC cells, protein expression levels were analyzed by Western blot analysis. In NSCLC cell lines, treatment with LPS and ATP significantly increased the expression levels of NLRP3 inflammasome proteins, including NLRP3 and ASC. Additionally, the activation of caspase-1, measured as the pro-caspase-1 (p50)/cleaved-caspase-1 (p20) ratio, also significantly increased in NSCLC cell lines stimulated with LPS and ATP compared to the non-stimulated group (*p* < 0.001), as shown in Figure 9. However, treatment with the morin compound significantly decreased the expression levels of NLRP3 and ASC and significantly reduced the pro-caspase-1 (p50)/cleaved-caspase-1 (p20) ratio, thereby decreasing caspase-1 activation in LPS+ATP-stimulated NSCLC cells in a dose-dependent manner (*p* < 0.001) (Figure 9A,B, Supplementary Materials Figures S6 and S7). Our results suggest that morin is associated with anti-inflammatory effects in LPS+ATP-stimulated NSCLC cells through suppressing the expression of NLRP3 inflammasome-related proteins.

### 3.8. Effects of Morin on the MAPK Signaling Pathway in LPS+ATP-Stimulated NSCLC Cells

**Figure 10:**
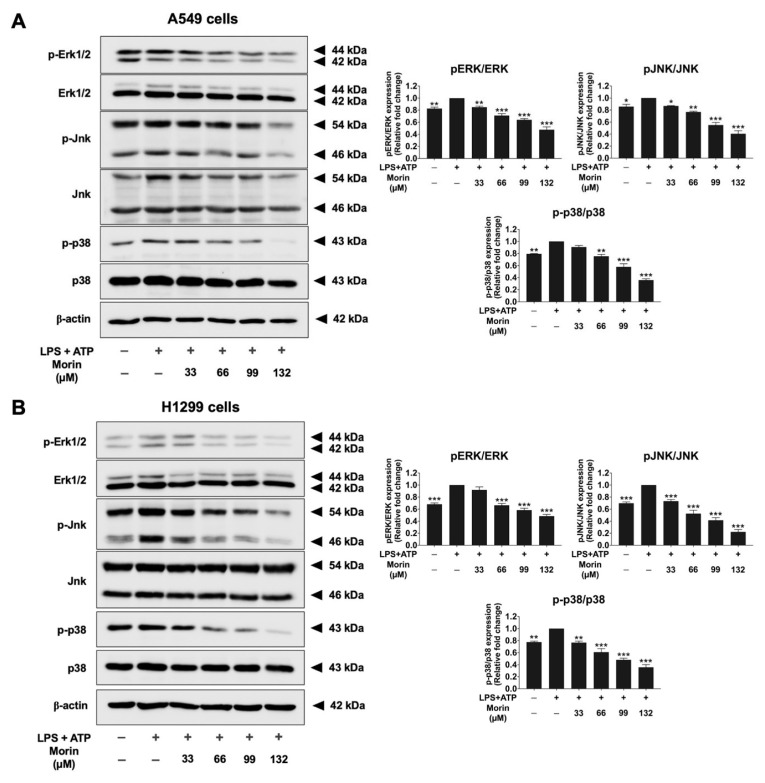
The suppressive effects of morin on the MAPK signaling pathway (ERK/JNK/p38) in LPS+ATP-stimulated NSCLC cells: A549 (**A**) and H1299 (**B**) cells. NSCLC cells were treated with morin at doses ranging from 0 to 132 μM for 24 h. Following treatment, the cells were stimulated with LPS (1 mg/mL) for 6 h, followed by stimulation with ATP (5 nM) for an additional 30 min. Western blotting and band density measurements were used to analyze the effects of morin on the expression of MAPK signaling pathway components (ERK, JNK, and p38) in NSCLC cells. These data are presented as percentages relative to the LPS+ATP-stimulated group, which is defined as 100%. These results are reported as the mean ± standard deviation, with * *p* < 0.05, ** *p* < 0.01, and *** *p* < 0.001, compared to the control group (LPS+ATP-stimulated group). Original Western blot images can be found in Figures S8 and S9.

To explore the properties of morin in the upstream regulatory pathways involved in NLRP3 inflammasome activation and NSCLC metastasis in LPS+ATP-stimulated A549 and H1299 cells, the phosphorylation of key signaling molecules in the MAPK signaling pathway (ERK, JNK, and p38) was investigated by Western blotting. In NSCLC cells, LPS+ATP treatment significantly stimulated the phosphorylation of key molecules in the MAPK signaling pathway, including ERK, JNK, and p38 proteins, compared to in the non-stimulated group (*p* < 0.001) of NSCLC cells, as shown in Figure 10. Conversely, morin treatment led to a significant and dose-dependent suppression of the phosphorylation levels of ERK, JNK, and p38 compared to in the LPS+ATP-stimulated group (*p* < 0.001) (Figure 10A,B, Supplementary Materials Figures S8 and S9). Our results suggest that morin may attenuate LPS+ATP-stimulated NLRP3 inflammasome activation by inactivating the ERK/JNK/p38 axis, leading to the reduced secretion of IL-6, IL-1β, and IL-18 pro-inflammatory cytokines.

## 4. Discussion

This study explored the anti-inflammatory and anti-metastatic properties of morin in non-small-cell lung cancer (NSCLC) cells, focusing on its effects on the NLRP3 inflammasome and MAPK signaling pathways. These effects resulted in the reduced production of pro-inflammatory cytokines (IL-1β, IL-18, IL-6), the suppression of cell migration and invasion, and the downregulation of EMT markers.

The LPS+ATP-induced inflammation and metastasis model is not cell-specific. Numerous studies have demonstrated the activation of the NLRP3 inflammasome by LPS+ATP in diverse cell types, including THP-1 macrophages [35], LX-2 hepatic stellate cells [36], MCF-12A and MDA-MB-231 breast cancer cells [37], and A549 non-small-cell lung cancer cells [16]. Using this well-established two-step priming model, we explored the anti-inflammatory and anti-metastatic properties of morin in the A549 and H1299 NSCLC cell lines. Pre-treating cells with morin ensures that the compound reaches a steady state before LPS+ATP induction. This approach is supported by prior studies that have demonstrated the efficacy of pre-treatment in mitigating inflammation and related pathways. For example, cytokine expression (IL-6, IL-1β, and IL-18) and NLRP3 inflammasome activation are significantly upregulated in A549 cells within 6 h of LPS+ATP induction. Pre-treatment with morin enables the compound to exert its full inhibitory effect, effectively blocking downstream pathways before LPS+ATP induce activation, thereby enhancing the reliability of our findings [26,27,38,39]. All candidate inflammatory gene expression levels (IL-6, IL-1β, IL-18, and NLRP3) and cytokine release levels (IL-6, IL-1β, and IL-18) in A549 cells were upregulated 6 h after LPS treatment followed by ATP stimulation for 30 min. This approach aligns with previous studies that employed short induction timeframes (3–8 h) to assess anti-inflammatory and anti-metastatic properties at both the gene and protein levels, with treatment with LPS+ATP for durations such as 3 h [35,40], 6 h [26,41,42], and 8 h [43] adjusted based on cell line aggressiveness. Based on this, pre-treating cell lines with LPS+ATP for at least 6 h and 30 min is necessary to effectively induce pro-inflammatory expression at both the gene and protein levels in LPS+ATP-induced cells. Furthermore, we selected a 6 h and 30 min timeframe for LPS+ATP induction to ensure consistency with other experiments assessing morin’s anti-inflammatory properties. This standardized timeframe allowed us to reliably observe the effects of morin on LPS+ATP-induced NSCLC cells.

The NLRP3 inflammasome is a crucial component of the innate immune response and has been implicated in cancer-related inflammation, contributing to tumor growth and metastasis [43,44,45]. In our study, LPS+ATP stimulation significantly elevated the expression of NLRP3, ASC, pro-caspase-1, and cleaved-caspase-1, leading to the increased secretion of pro-inflammatory cytokines IL-1β and IL-18. These findings are consistent with previous reports highlighting the role of the NLRP3 inflammasome in enhancing the pro-tumorigenic microenvironment in lung cancer [30,43]. Our findings demonstrate that morin does not exert cytotoxic effects on NSCLC cells, including both A549 and H1299 cells, under the tested conditions. Although previous studies have reported that morin can inhibit cell proliferation and migration [46] at a high concentration (600 uM), our SRB assay revealed no significant changes in cell viability after 48 h of treatment. Both cell lines showed comparable viability to the control, indicating that at a concentration of 165 µM and within a 48 h timeframe, morin does not interfere with cell proliferation or induce cytotoxicity. This supports its use in subsequent studies exploring its biological effects without confounding factors, such as proliferation inhibition or cell death. Additionally, we acknowledge that morin has been reported to inhibit proliferation in other cancer models. These effects may depend on the concentration [46] as well as the specific cell line, as demonstrated in bladder cancer cells [46] and in CML cells [47]. However, in our study, morin showed no adverse effects on cell viability within the experimental conditions employed. On the other hand, other reports at the same concentration of morin with the same timeframe duration showed no cytotoxicity, which is similar to our findings [48,49]. Morin treatment significantly downregulated the expression of NLRP3 inflammasome components in a dose-dependent manner, thereby reducing the levels of IL-1β and IL-18. This suggests that morin may effectively inhibit inflammasome activation, potentially disrupting the inflammatory cascade that promotes NSCLC metastasis. While previous studies have shown the anti-inflammatory properties of morin in various diseases [18,50,51], our findings are the first to demonstrate its inhibitory effects on the NLRP3 inflammasome in NSCLC, highlighting its potential as a novel anti-cancer agent.

The MAPK pathway, involving ERK, JNK, and p38 signaling, plays a pivotal role in regulating cellular responses such as proliferation, differentiation, and migration [52]. Dysregulation of this pathway is commonly observed in NSCLC, contributing to enhanced tumor invasion and metastasis [53,54]. In our study, we selected a 6 h and 30 min timeframe for LPS+ATP induction to assess the inhibitory effects of morin on the MAPK signaling pathway while maintaining consistency with other experiments. This timeframe is consistent with a previous study conducted in RAW264.7 murine macrophage cells [42] using similar conditions to detect the phosphorylation of the same proteins assessed in the present study. In our experiments, LPS+ATP stimulation significantly increased the phosphorylation of ERK, JNK, and p38 proteins, indicating activation of the MAPK pathway in A549 and H1299 cells. Morin treatment markedly suppressed the phosphorylation of ERK, JNK, and p38, suggesting its potential to inhibit MAPK pathway activation. The inhibition of the MAPK pathway by natural compounds like morin has been highlighted in recent cancer studies [55,56], suggesting its potential as an adjuvant therapeutic strategy to reduce tumor metastasis and inflammation. By blocking this pathway, morin may prevent the transcription of genes involved in inflammatory responses and cell migration, thereby reducing the metastatic potential of NSCLC cells.

Metastasis remains a major challenge in the treatment of NSCLC, with epithelial-to-mesenchymal transition (EMT) playing a critical role in enhancing the migratory and invasive capabilities of cancer cells [57]. Our data indicated that LPS+ATP induction significantly modifies the protein expression of invasive proteins (MMP-2, MMP-9, u-PAR, u-PA, and MT1-MMP) and mesenchymal markers (fibronectin, N-cadherin, and vimentin). Additionally, our results showed that morin significantly reduced the expression of invasive proteins and mesenchymal markers in LPS+ATP-induced NSCLC cells. These proteins are critical for facilitating cell metastasis. In addition, it is well established that the A549 and H1299 cells are highly metastatic NSCLC cell lines with consistent expression of mesenchymal markers [58]. Our findings align with prior studies that demonstrate that the suppression of mesenchymal markers correlates with reduced tumor metastasis [58,59,60,61]. The downregulation of these markers suggests that morin disrupts the processes involved in cell invasion and migration, likely through its inhibitory effects on the NLRP3 inflammasome and MAPK signaling pathways. By reducing the activation of these pathways, morin may hinder mesenchymal marker expression and prevent the degradation of components in the extracellular matrix, with these processes being essential steps in the invasion and metastasis process of cancer. These findings are consistent with previous research indicating that the suppression of EMT markers is associated with decreased tumor metastasis and better clinical outcomes in patients with NSCLC [57,62,63].

The dual inhibitory effects of morin on inflammation and metastasis highlight its potential as a promising therapeutic agent for NSCLC. Unlike conventional chemotherapy, which often targets rapidly dividing cells non-selectively, morin appears to specifically target the inflammatory pathways that drive tumor progression. This specificity could translate into a more effective treatment strategy with fewer side effects, especially when used in combination with existing therapies. Given its low toxicity and natural origin, morin could be a viable alternative for long-term treatment, with the aim of suppressing chronic inflammation and metastasis spread in patients with NSCLC. Future studies should explore the pharmacokinetics and bioavailability of morin in vivo to confirm its therapeutic potential and evaluate its efficacy in combination with standard chemotherapy agents.

While our in vitro findings are promising, there are several limitations to consider. First, the effects of morin were evaluated in established NSCLC cell lines, which may not fully represent the tumor microenvironment and its complexity in vivo. Therefore, further studies using animal models and patient-derived xenografts are needed to validate the therapeutic efficacy of morin in NSCLC. Additionally, detailed pharmacokinetic studies are required to assess the absorption, distribution, metabolism, and excretion of morin, which will be critical for its clinical development. Finally, exploring the synergistic effects of morin with other anti-cancer agents, for example, immune checkpoint inhibitors or targeted kinase inhibitors, could provide valuable insights into its potential role in combination therapies for NSCLC.

## 5. Conclusions

In conclusion, this study provides strong evidence that morin exerts anti-inflammatory and anti-migratory effects in LPS+ATP-stimulated NSCLC cells through the inhibition of the NLRP3 inflammasome and MAPK signaling pathways. These findings support the idea that morin could serve as a novel therapeutic strategy to counteract inflammation-driven metastasis in NSCLCL, offering a potential avenue for improving patient outcomes in this highly metastatic cancer.

## Data Availability

Data are contained within the article and the Supplementary Materials.

## Supplementary Materials

The following supporting information can be downloaded at: https://www.mdpi.com/article/10.3390/biom15010103/s1, Figure S1: The anti-migration effect of morin on NSCLC cells, A549 and H1299 cells; Figure S2: Original blots of morin suppressed the invasive protein (u-PAR, u-PA, MT1-MMP) in LPS+ATP-induced A549 cells; Figure S3: Original blots of morin suppressed the invasive protein (u-PAR, u-PA, MT1-MMP) in LPS+ATP-induced H1299 cells; Figure S4: Original blots of morin suppressed EMT markers (Fibronectin, N-cadherin, and Vimentin) in LPS+ATP-induced A549 cells; Figure S5: Original blots of morin suppressed EMT markers (Fibronectin, N-cadherin, and Vimentin) in LPS+ATP-induced H1299 cells; Figure S6: Original blots of morin suppressed the NLRP3 inflammasome pathway (NLRP3, ASC, pro-caspase-1 and cleaved-caspase-1 proteins) in LPS+ATP-induced A549 cells; Figure S7: Original blots of morin suppressed the NLRP3 inflammasome pathway (NLRP3, ASC, pro-caspase-1 and cleaved-caspase-1 proteins) in LPS+ATP-induced H1299 cells; Figure S8: Original blots of morin inactivated the MAPK signaling pathway (ERK/JNK/p38) in LPS+ATP-induced A549 cells; Figure S9. Original blots of morin inactivated the MAPK signaling pathway (ERK/JNK/p38) in LPS+ATP-induced H1299 cells.
