# Supplementary material for: Anti-Inflammatory and Anti-Migratory Effects of Morin on Non-Small-Cell Lung Cancer Metastasis via Inhibition of NLRP3/MAPK Signaling Pathway
Source: Biomolecules. 2025 Jan 10;15(1):103. doi: 10.3390/biom15010103 (PMC11763329; doi:10.3390/biom15010103)
Supplement: Supplementary file 1 [file biomolecules-15-00103-s001.zip › biomolecules-3353503-supplementary.docx]

Supplementary Materials


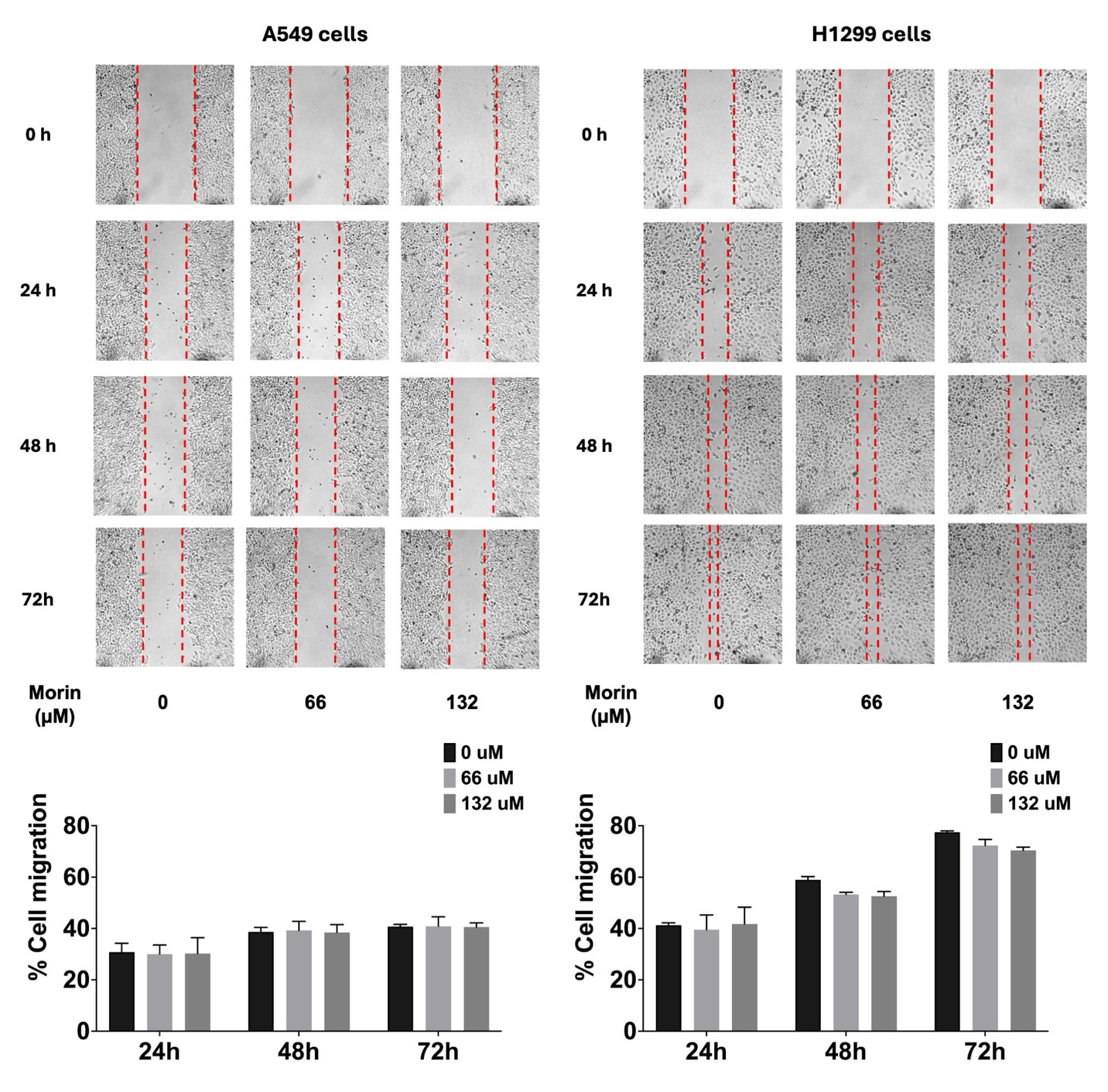


**Figure S1.** The anti-migration effect of morin on NSCLC cells, A549 and H1299 cells. The anti-migration properties of morin (0, 66, and 132 μM) were evaluated using a scratch assay. The images captured after the initial scratch at 0-, 24-, 48- and 72-hours. Migrated cells were visualized using phase-contrast microscopy and quantified with ImageJ software. These results are reported as the mean ± standard deviation.


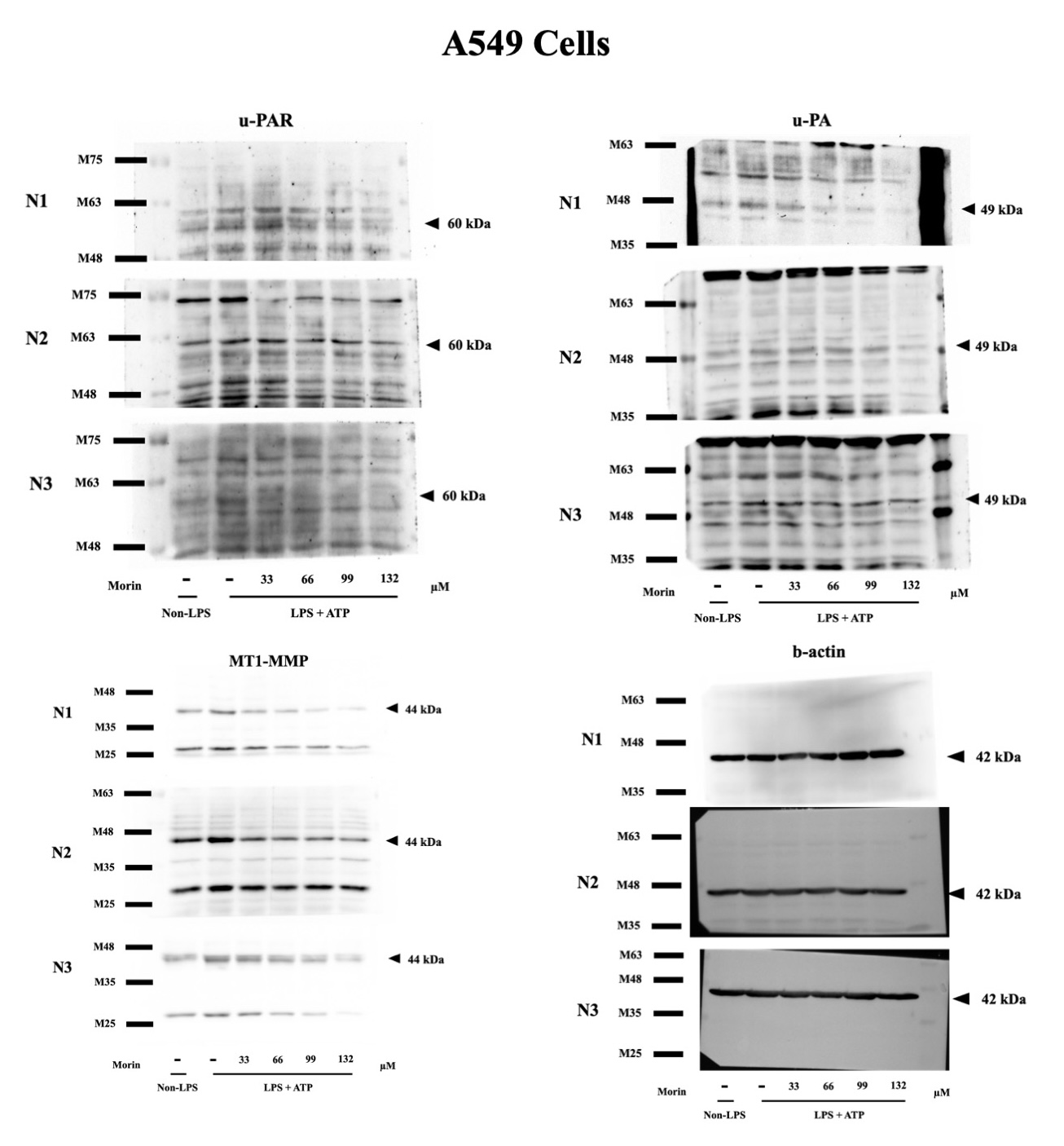


**Figure S2.** Original blots of morin suppressed the invasive protein (u-PAR, u-PA, MT1-MMP) in LPS+ATP-induced A549 cells.


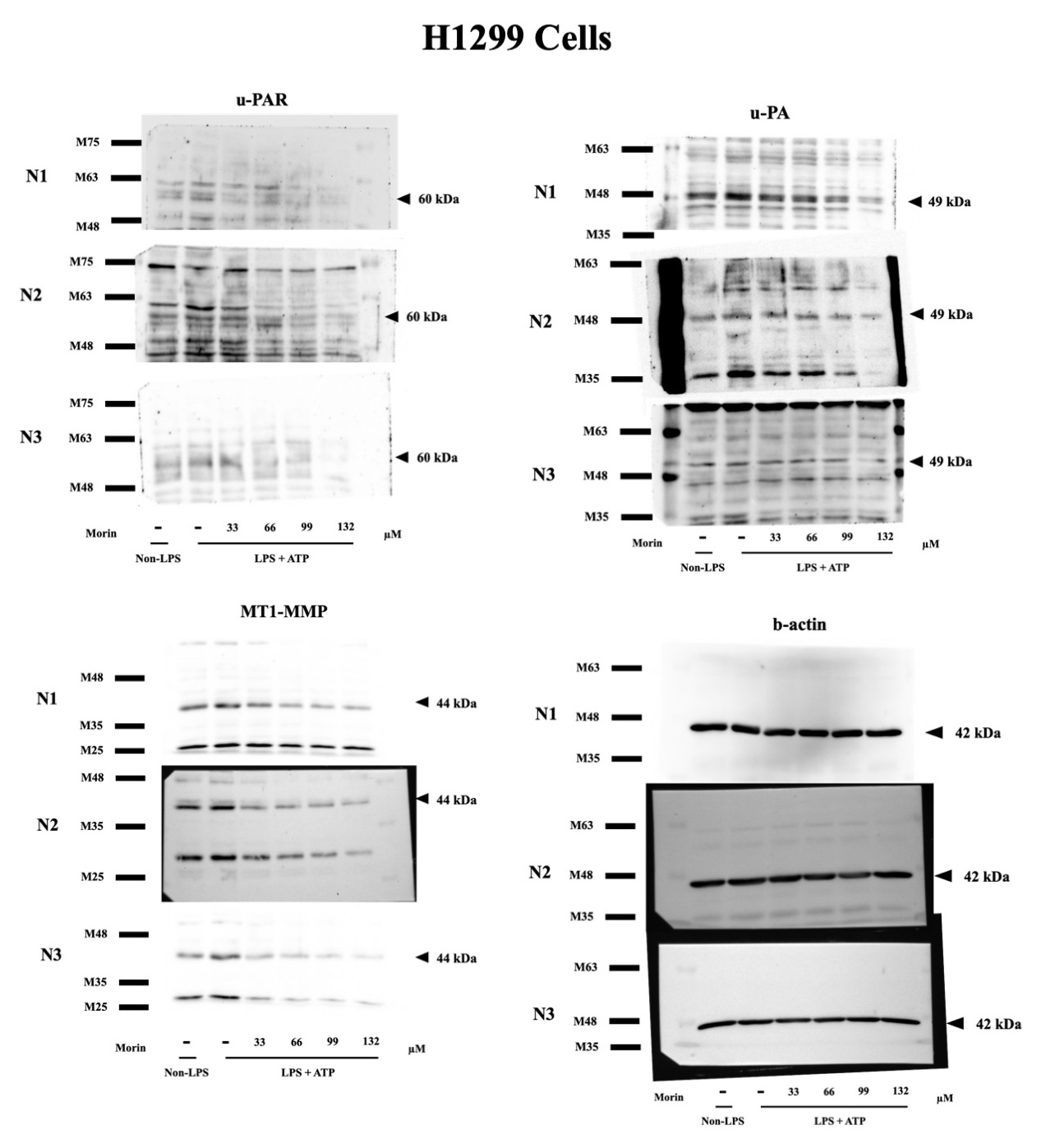


**Figure S3.** Original blots of morin suppressed the invasive protein (u-PAR, u-PA, MT1-MMP) in LPS+ATP-induced H1299 cells.


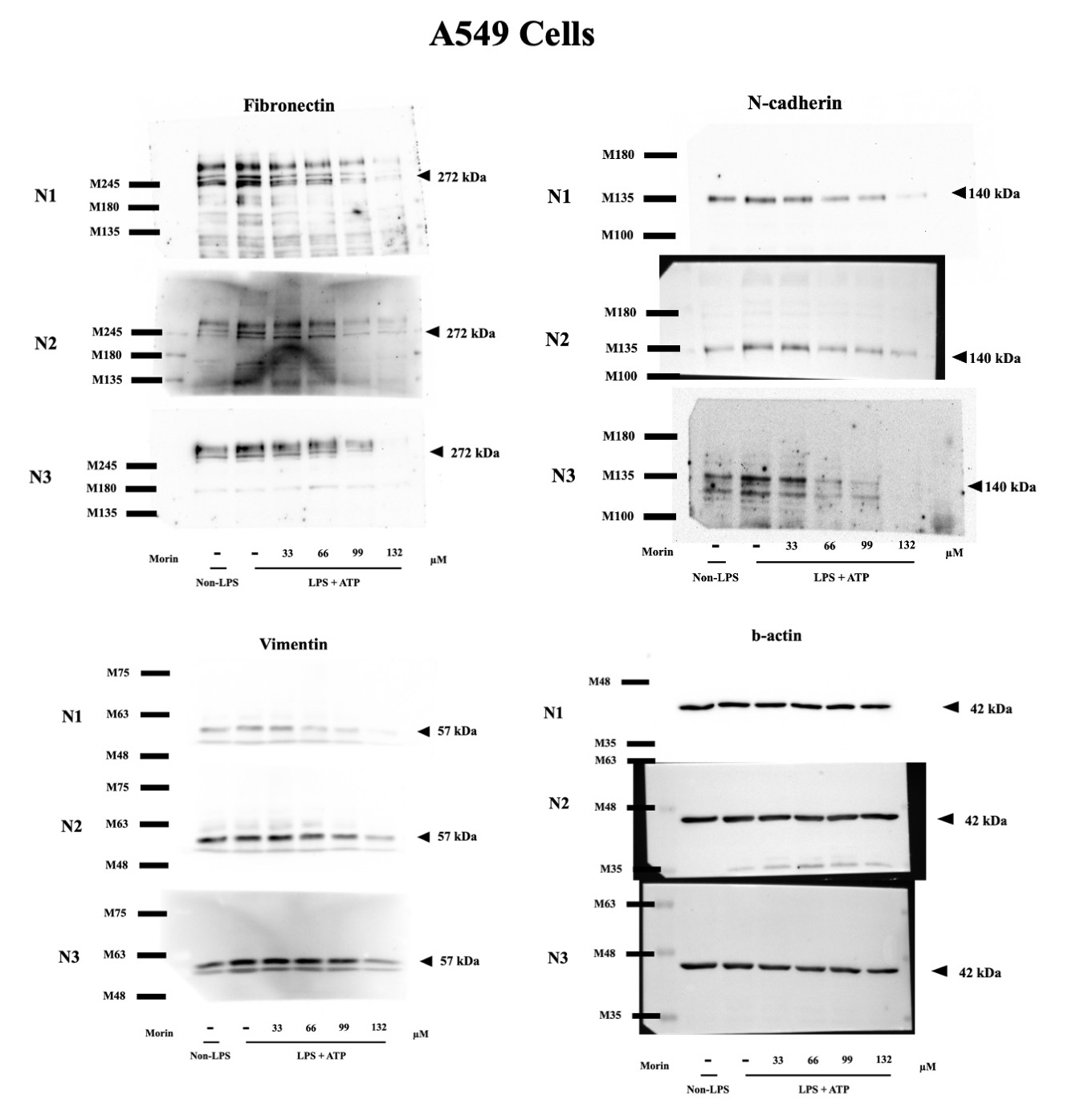


**Figure S4.** Original blots of morin suppressed EMT markers (Fibronectin, N-cadherin, and Vimentin) in LPS+ATP-induced A549 cells.


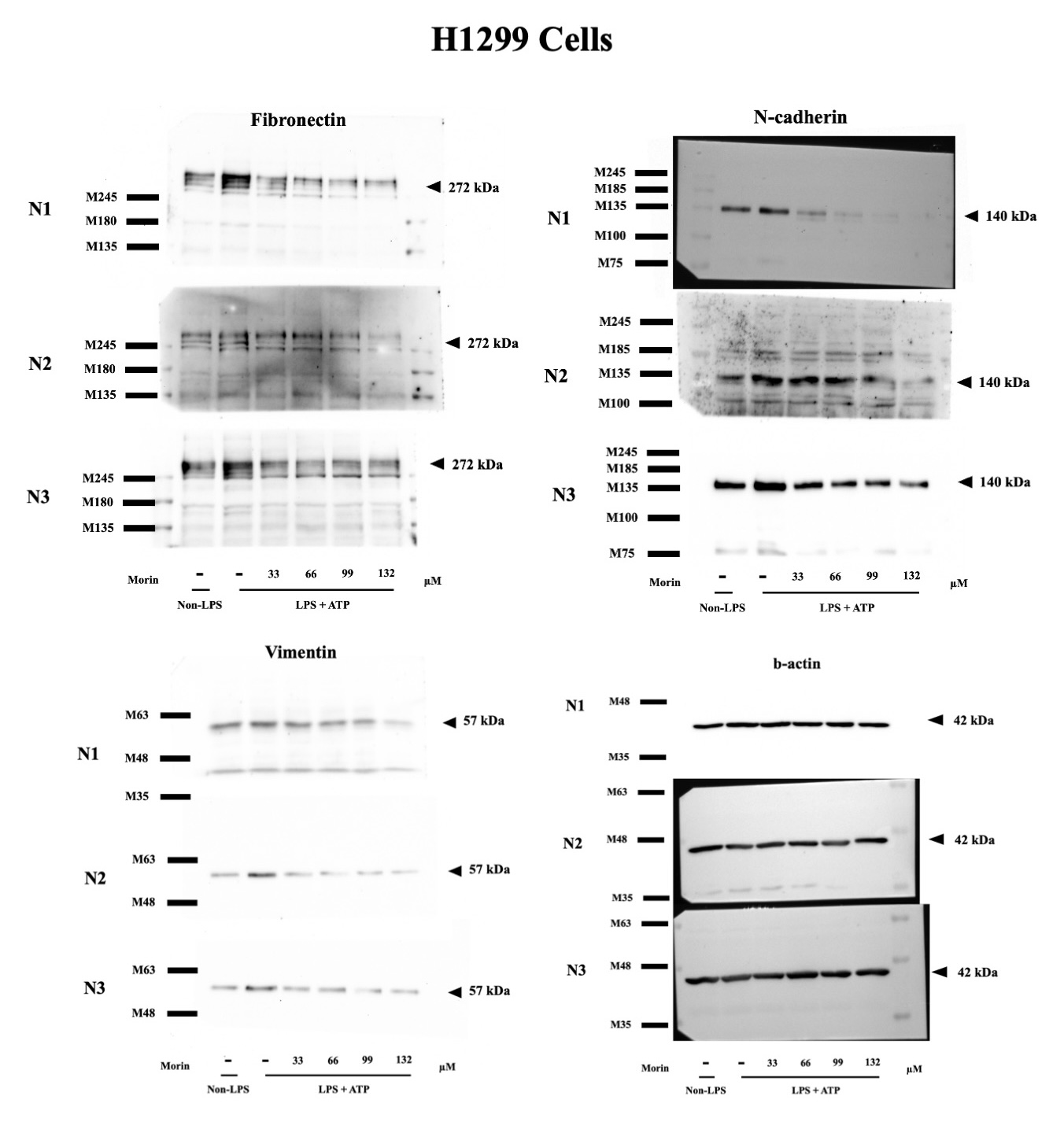


**Figure S5.** Original blots of morin suppressed EMT markers (Fibronectin, N-cadherin, and Vimentin) in LPS+ATP-induced H1299 cells.


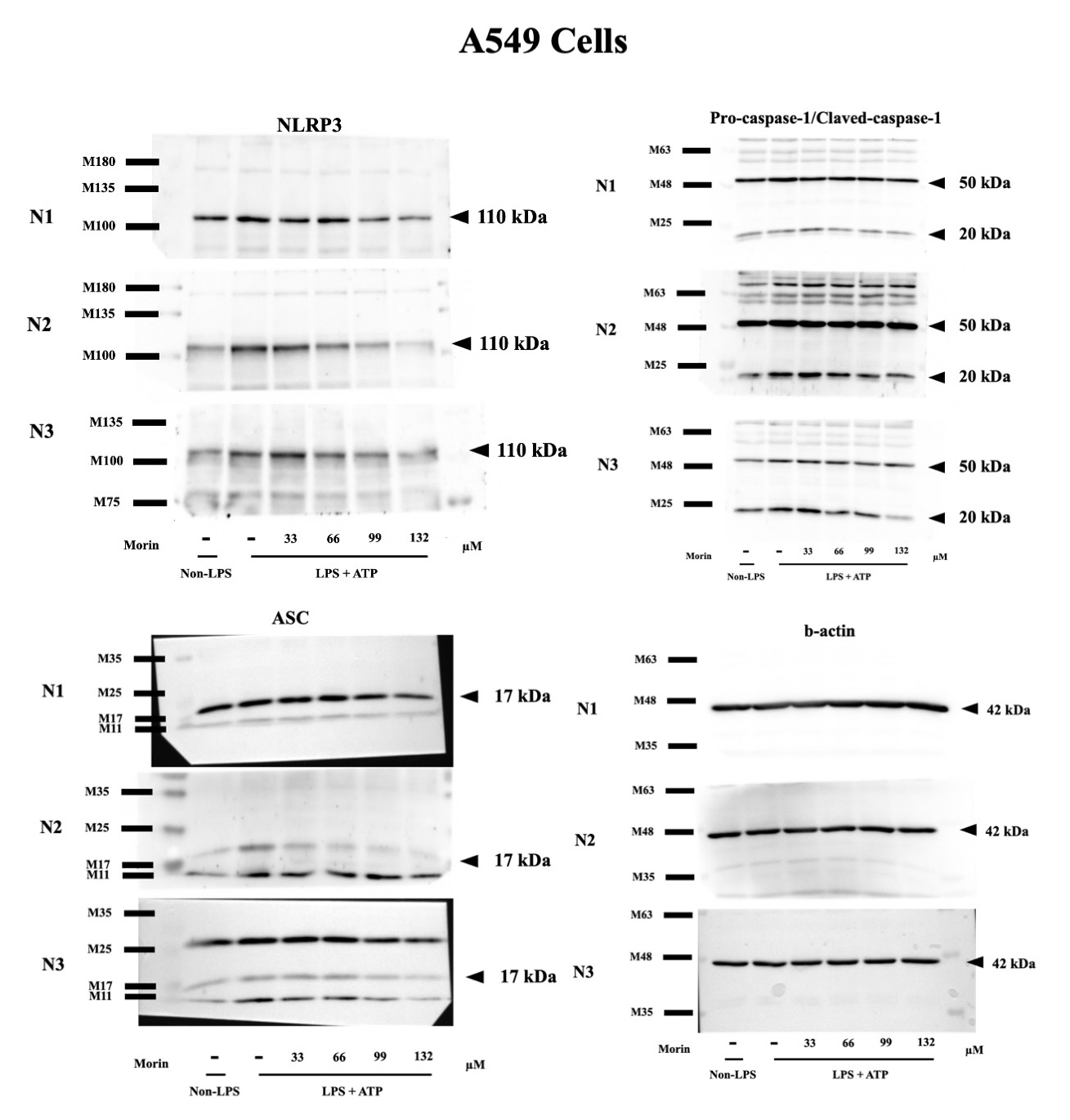


**Figure S6.** Original blots of morin suppressed the NLRP3 inflammasome pathway (NLRP3, ASC, pro-caspase-1 and cleaved-caspase-1 proteins) in LPS+ATP-induced A549 cells.


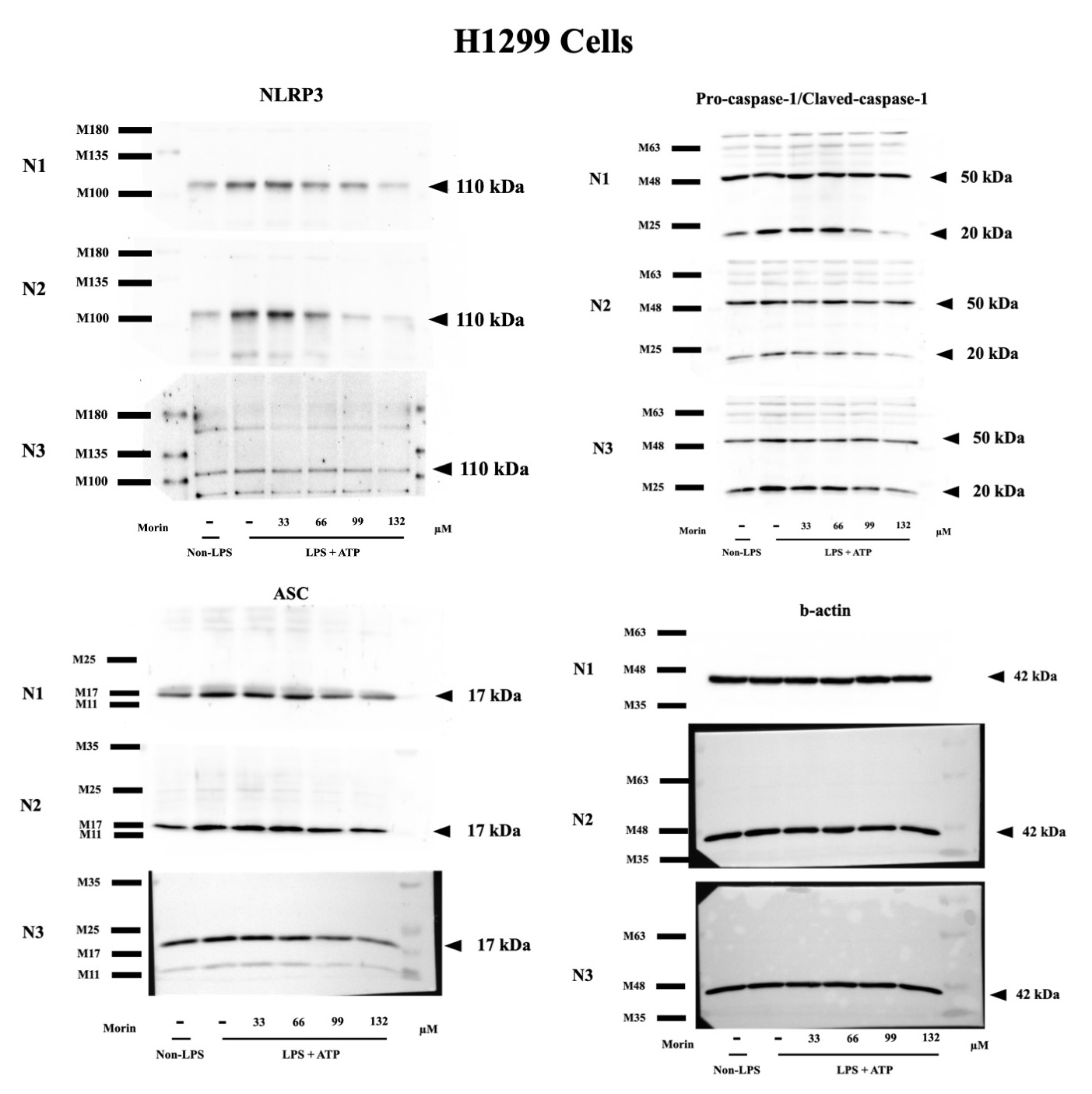


**Figure S7.** Original blots of morin suppressed the NLRP3 inflammasome pathway (NLRP3, ASC, pro-caspase-1 and cleaved-caspase-1 proteins) in LPS+ATP-induced H1299 cells.


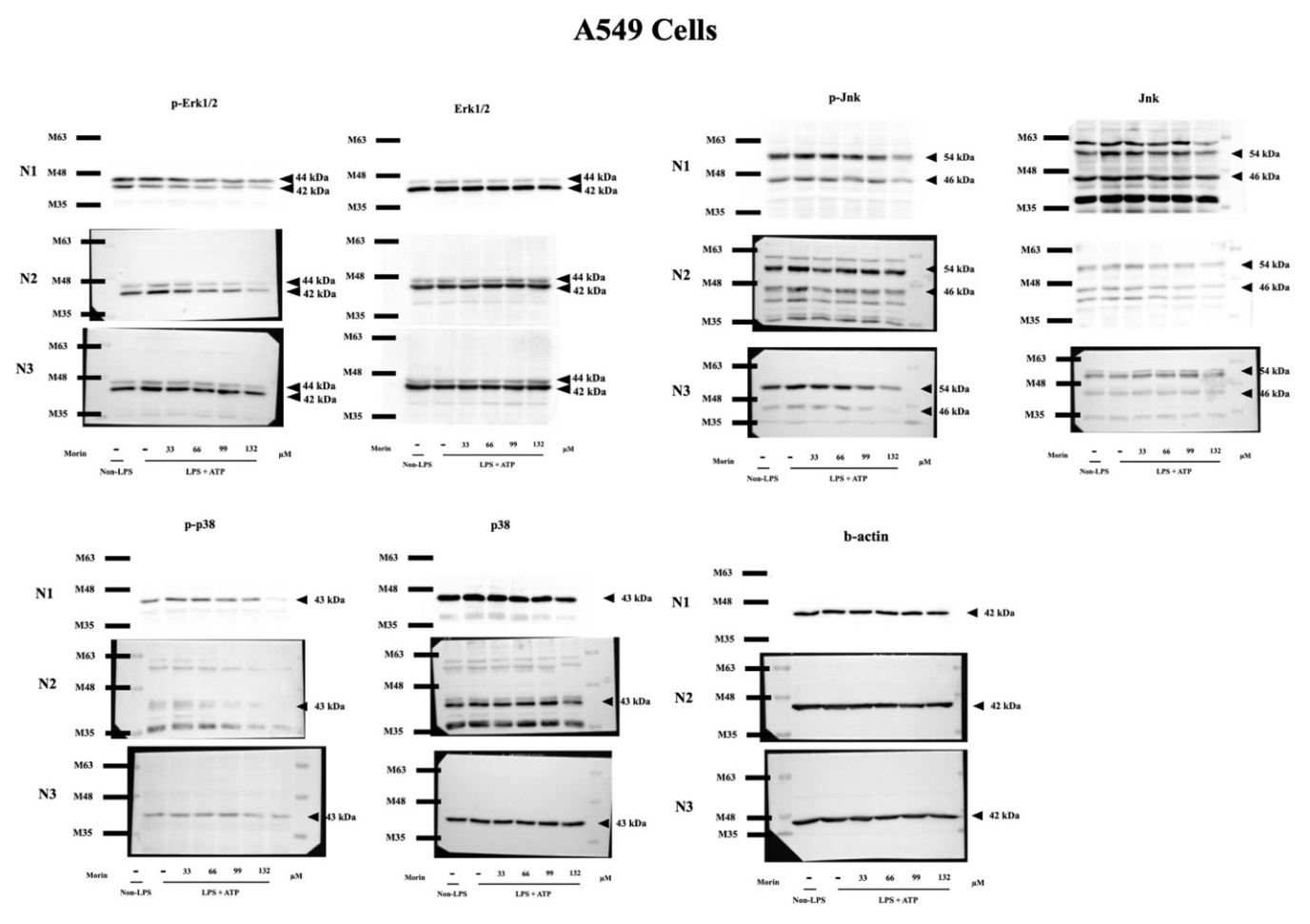


**Figure S8.** Original blots of morin inactivated the MAPK signaling pathway (ERK/JNK/p38) in LPS+ATP-induced A549 cells.


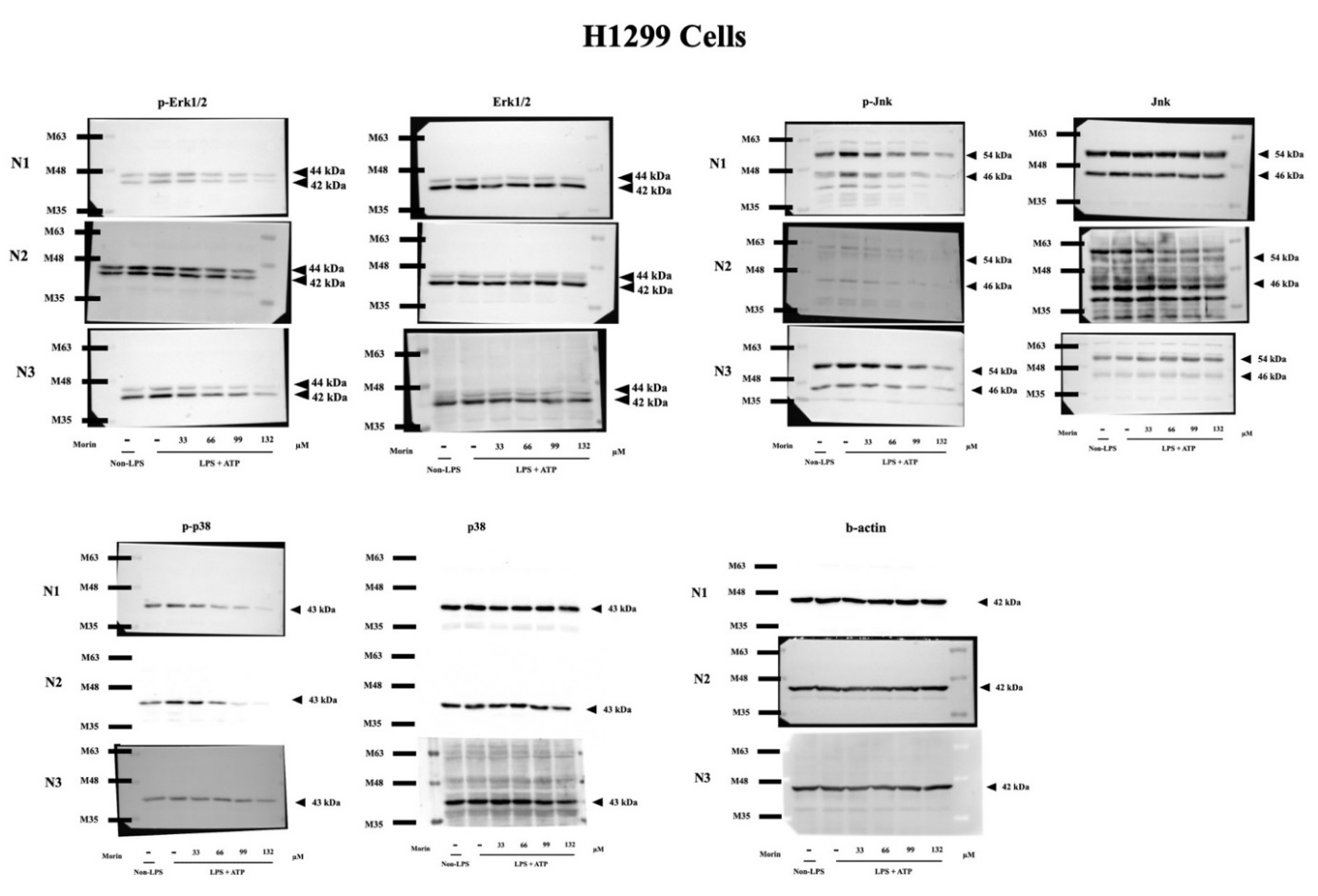


**Figure S9.** Original blots of morin inactivated the MAPK signaling pathway (ERK/JNK/p38) in LPS+ATP-induced H1299 cells.
